# Death from a Drop of Laudanum

**Published:** 1849-11

**Authors:** 


*Death from a Drop of Laudanum.* By H. V. WOOTEN, M. D., of Lowndesboro, Alabama.

A fine, healthy, female child, in the 5th day of its age, suffered from "griping," as its mother supposed, for which she administered to it *one drop* of laudanum. Thirty minutes afterwards, its breathing becoming slow and stertorous, I was sent for; but being absent, another physician saw it, who found it impossible to get the child to swallow any thing. External excitants, etc., were resorted to, and three hours after the laudanum was taken I saw it. Its pupils were dilated and insensible to light, breathing very laborious, each inspiration giving a loud, struggling sound, great lividity of complexion, etc. It would draw four inspirations, at the rate of sixteen per minute, and then cease to inhale about thirty seconds, when the four inspirations would again be drawn. On the fourth inspiration, a general spasm of the extremities would seize it. Its pulse during the last two inspirations were about fifty to the minute, during the spasm and suspension of breathing it would run up to about 100, become very weak, and finally cease at the wrists about six seconds before the breathing was resumed.

This condition continued without material variation until the sixth hour, when on bathing it in hot water and brandy, followed by the application of plasters of cayenne to the feet and hands, it breathed, continuously, but with great difficulty, at the rate of 30 inspirations to the minute, for twenty minutes, and its pulse during this time ranged from 90 to 100. Its pupils contracted a little, and the lividity of complexion disappeared to a considerable extent. Hopes were now entertained that it had passed the crisis, and would recover; but spasms again seized it, from which it fell into a collapse, from which nothing that we could do would raise it. After this it would draw only three inspirations at the rate of twelve to the minute, when spasms would occur, and the suspensions of breathing become longer. At the tenth hour, it drew but two inspirations together, about twelve seconds apart, and then suspend for nearly a minute. For three hours, I thought during every suspension of breathing, that it was dead, as its pulse would cease at the wrists before breathing was resumed; but it continued to labor for breath in this way until the end of the eleventh hour, when it died.

The laudanum was dropped from an ounce vial, in which there was but about ten drops. It had been stopped with a piece of twisted paper, and hanging up about a year; all the inner surface of the lower part of the vial was encrusted with opium, and the remaining laudanum was heavily charged with this deposit resulting from evaporation. Every means of keeping the child alive, which our ingenuity could suggest, were diligently applied, and with apparent effect, but not success.

This case is one which rarely occurs, and I report it mainly on that account; yet it is not otherwise destitute of interest. The stomach pump was not used, because I had no tube of suitable size, and besides, I was satisfied that it was too late to resort to measures of that kind when I saw it. *Southern Med. and Surg. Journal.*

